# SEX DIFFERENCES AND LONGITUDINAL QUANTILES OF FRAILTY TRAJECTORIES WITH MISSING DATA DUE TO DEATH

**DOI:** 10.1093/geroni/igac059.2997

**Published:** 2022-12-20

**Authors:** Alejandra Marroig, Fernando Massa, Scott Hofer, Graciela Muniz Terrera

**Affiliations:** Universidad de la Republica Uruguay, Montevideo, Montevideo, Uruguay; Universidad de la República, Montevideo, Montevideo, Uruguay; University of Victoria, Victoria, British Columbia, Canada; Ohio University, Athens, Ohio, United States

## Abstract

Scarce evidence exists about frailty trajectories, but the evidence suggests that women live longer with higher levels of frailty. When progression of frailty was studied, the focus has been on mean trajectories and research has ignored death. Here, we aim to assess the role of sex, age, and education in different quantiles of the distribution of frailty trajectories. We derived a frailty index (FI, range 0–1) based on the accumulation of deficits in individuals aged 65 at baseline (n=6929) using data from the Survey of Health, Ageing, and Retirement in Europe (SHARE). We applied weighted Generalized Estimating Equations (weighted GEE) to adjust the quantiles of the FI trajectory by sex, and education. The results show that the median FI trajectory increases with age (b_age0.5=0.008, p < 0.001) and this increase is higher for women than men (b_age*sex0.5=0.003, p < 0.001), but sex differences disappear for the most frail (0.9 percentile) once the missing data process is accounted for (b_age*sex0.9=0.002, p=0.085). For the most frail the increase with age is higher than for those at the median (b_age0.9=0.018, p < 0.001; test diff, p < 0.001) and education reduces the progression of the median FI (b_age*edu0.5=-0.0003, p < 0.001). Our approach advances the understanding of frailty trajectories of older adults, showing differences across quantiles. Thus, this may improve the practice and design of interventions aimed at older adults. In addition, we address the process of missing information and the results show new insights, particularly for those who are most frail.

